# Liberation from Mechanical Ventilation in Acute Hypoxemic Respiratory Failure or Adult Respiratory Distress Syndrome: A Review

**DOI:** 10.3390/jcm15156019

**Published:** 2026-08-03

**Authors:** Karen E. A. Burns, Karen J. Bosma, Bruno L. Ferreyro, Dipayan Chaudhuri, Andrew J. E. Seely, Daniel R. Ouellette

**Affiliations:** 1 Department of Medicine, Interdepartmental Division of Critical Care, University of Toronto, Toronto, ON M5G 1V7, Canada; 2 Department of Medicine, Division of Critical Care, Unity Health Toronto—St. Michael’s Hospital, Toronto, ON M5B 1W8, Canada; 3 Faculty of Medicine, The University of Western Ontario, London, ON N6A 3K7, Canada; 4 Division of Critical Care, London Health Sciences Centre, London, ON N6A 5A5, Canada; 5 Department of Medicine, Sinai Health System, University of Toronto, Toronto, ON M5G 1V7, Canada; 6 Department of Critical Care, Division of Critical Care Medicine, Markham-Stoufville Hospital, Markham, ON L3P 7P3, Canada; 7 Department of Medicine, Division of Critical Care, McMaster University, Hamilton, ON L8S 4L8, Canada; 8 Acute Care Research, Ottawa Hospital Research Institute, The University of Ottawa, Ottawa, ON K1N 6N5, Canada; 9 Department of Critical Care, Ottawa Hospital—General Campus, Ottawa, ON K1H 8L6, Canada; 10 Division of Pulmonary and Critical Care Medicine, Henry Ford Health System, Detroit, MI 48202, USA

**Keywords:** weaning, mechanical ventilation, acute respiratory failure, acute respiratory distress syndrome, noninvasive ventilation

## Abstract

Efforts to liberate patients from invasive mechanical ventilation (MV) begin when the underlying cause of acute hypoxemic respiratory failure (AHRF) or adult respiratory distress syndrome (ARDS) that led to use of invasive ventilation has resolved or improved and patients can initiate spontaneous breaths. In preparation for liberation, clinicians transition patients to spontaneous modes of ventilation as soon as possible while ensuring that patients’ respiratory effort is not insufficient or excessive during weaning attempts. Concurrently, clinicians aim to minimize the effects of sedative and analgesic agents, screen daily to identify patients who are ready to undergo a spontaneous breathing trial (SBT), and conduct SBTs to help assess patients’ readiness for extubation. Extubation failure is rarely the consequence of a single physiological abnormality. Rather, it reflects the interaction of multiple mechanisms that often coexist, including an imbalance between respiratory system load and capacity, ineffective cough, and secretion burden among others. For these reasons, single weaning parameters and SBTs may fail to identify some patients who are at risk for extubation failure. Conversely, indices and scores that combine two or more parameters and newer techniques may provide mechanistic insights into the pathways that lead to extubation failure, help to characterize ‘at-risk’ phenotypes, and identify patients who may benefit from closer monitoring, targeted therapeutic strategies, and/or early application of noninvasive respiratory support strategies such as high-flow nasal cannula (HFNC) and bilevel noninvasive positive pressure ventilation (NIV).

## 1. Introduction

Liberation or weaning is the process during which the work of breathing is transferred from the ventilator back to the patient. Roughly 40% of the time spent on invasive mechanical ventilation is consumed by weaning [1]. Although invasive ventilation is effective, it is associated with the development of numerous complications, including respiratory muscle weakness, upper airway injury, ventilator-associated pneumonia (VAP) [2] and sinusitis [3]. The incidence of VAP increases exponentially after day 5 of invasive mechanical ventilation (MV) and has been associated with both increased morbidity and a trend toward increased mortality [4]. Accordingly, limiting the duration of invasive MV has been identified as a key research priority in critical care [5].

Timely and safe liberation (extubation) from invasive MV is a high-stakes decision. To reduce complication rates and improve outcomes, clinicians strive to shorten patients’ exposure to invasive MV. However, premature or failed attempts at extubation necessitating reintubation are associated with morbidity (e.g., VAP) and an approximate 8- to 10-fold increase in mortality [6]. While attempting to limit a patient’s exposure to invasive MV, clinicians ‘trade-off’ the risks associated with premature failed attempts at extubation (too early) and the complications associated with continued or protracted invasive MV (too late). To address liberation for patients with acute hypoxemic respiratory failure (AHRF) and acute respiratory distress syndrome (ARDS), five experts in weaning, extubation, and post-extubation respiratory support worked in parallel to review and summarize the literature in this narrative review across five domains (one domain per expert) pertaining to identifying liberation candidates, ventilator modes and reducing support, conduct of spontaneous breathing trials (SBTs), extubation, and the use of noninvasive post-extubation respiratory support strategies.

## 2. Identifying Candidates for Ventilator Liberation

### 2.1. Assessing Readiness for Weaning

A general medical assessment to identify candidates for ventilator liberation begins with the observation that the patient is recovering from the initial condition leading to the need for invasive MV [5]. The patient should be clinically stable with acceptable hemodynamic parameters receiving no more than minimal doses of vasoactive agents [7,8]. Classically, patients were not considered for liberation from MV if they were receiving vasoactive agents, but studies have demonstrated that low stable doses of vasopressors may be acceptable [8]. In a large, retrospective cohort study, ventilator liberation while receiving low doses of vasopressor agents (<0.1 ug/kg/min) was associated with lower mortality, decreased intensive care unit (ICU) length of stay (LOS), and no difference in extubation failure rates compared to the rest of the study group. By contrast, mortality and extubation failure rates were increased in patients disconnected from MV while receiving high doses of vasopressors [8].

Several physiologic parameters should be met prior to considering MV liberation, including the following items. An intact respiratory drive must be present. This can be determined by observing patient-triggered breaths on a ventilator supportive mode allowing spontaneous triggering or by briefly placing patients on a spontaneous mode and observing patient breath initiation. The patient should have acceptable gas exchange as indicated by an arterial partial pressure of oxygen (P_a_O_2_) > 60 mm Hg or arterial oxygen saturation (S_a_O_2_) > 90% on an F_i_O_2_ < 0.5 and positive end-expiratory pressure (PEEP) < 10 cm H_2_O, and a P_a_CO_2_ = ~40 mm Hg or consistent with a chronic, stable respiratory acidosis. [7,9]. A neurologic examination assessing cognition, muscle strength, and cranial nerve function should be performed [10], and the patient should be able to generate a cough [11].

### 2.2. Weaning Prediction

The likelihood of successful liberation from MV may be informed by the rapid shallow breathing index (RSBI). Yang and Tobin assessed several indexes first in a training cohort and then in a prospective validation cohort to determine the predictive accuracy of each for ventilator liberation outcomes [12]. The RSBI in this study was determined by discontinuing MV, allowing the patient to breathe room air for one minute, and then determining the ratio between the respiratory frequency and tidal volume. A threshold value < 105 breaths/min/L was determined to be more predictive of successful liberation outcomes than other measurements. Prospective clinical trials subsequently demonstrated the utility of SBTs in the assessment of candidates for MV liberation [13]. Vassilakopoulos and co-workers studied the determinants of liberation at the end of a T-piece trial and suggested that RSBI, when determined at the end of the SBT, was highly associated with successful discontinuation of MV [14]. Although the use of the RSBI at the end of an SBT to assess a patient for successful MV liberation was recommended by an early clinical practice guideline, experts noted that the positive likelihood ratio (1.49) for the association of the RSBI with successful liberation was modest in magnitude and suggested that other factors needed to be considered as part of a global assessment of liberation readiness [5]. Subsequently, a meta-analysis of 48 studies identified that although the sensitivity of the RSBI for predicting extubation success was high (83%), the specificity was modest (58%) and did not vary with use of different thresholds (<80 versus 80–150 versus <105 breaths/min/L), the support used during RSBI measurement, or the timing of RSBI measurement [15].

### 2.3. Daily Assessments

Once a patient has been identified as being a potential candidate for liberation from MV, daily assessments should be performed to determine readiness to liberate. Variation in practice is reduced and improved clinical outcomes are realized by the use of protocolized approaches to daily ventilator liberation assessment. Ely and colleagues randomized 300 patients to a daily two-step protocol of screening followed by an SBT compared to a usual care cohort where patients received screening alone. Patients in the protocolized arm had a median duration of MV of 4.5 days compared to 6 days in the usual care arm [16]. Kollef evaluated a similar protocol administered by nursing and respiratory therapy personnel and demonstrated improvements in the duration of MV [17].

More recent randomized controlled trials (RCTs) have evaluated different timing and frequency of screening, as well as different criteria to conduct SBTs. Hernández Martínez et al. found that the combination of earlier screening [initiated when the PaO_2_/(fractional concentration of oxygen) FiO_2_ > 180 mmHg] with more aggressive SBTs (conducted with PEEP 10 cmH_2_O) was associated with a higher reintubation rate [18]. Burns and coworkers evaluated both screening frequency (once versus more frequent) and SBT techniques [pressure support (PS) (>0 to 8 cm H_2_O) + PEEP (>0 to 5 cm H_2_O) versus T-piece] for patients who received invasive MV for more than 24 h. In the main analysis, neither screening frequency nor SBT technique influenced the time to successful extubation. Pairwise contrasts revealed two key findings: (i) when PS SBTs were used, more frequent screening (versus once-daily screening) increased the time to successful extubation, and (ii) when once-daily screening was used, PS + PEEP (versus T-piece) SBTs shortened the time to first successful SBT [19]. Findings from these trials caution against the combined use of early aggressive screening criteria and SBT settings and more frequent screening when PS SBTs are conducted.

Strategies to minimize the effects of sedative and narcotic agents on cognition and airway protective reflexes such as cough are important to consider in preparing the patient to be liberated from invasive ventilation [20,21,22]. Kress and colleagues prospectively studied 128 patients, randomizing them to either receive daily interruptions of sedative medications versus continuous infusion of sedatives. Daily interruptions of sedation reduced the duration of MV by more than 2 days and reduced ICU LOS by 3.5 days [23]. Clinical studies comparing cohorts of mechanically ventilated patients managed with protocols limiting sedatives compared to those receiving usual care consistently demonstrated that protocolized sedation management leads to shorter duration of ventilation [24,25].

The pairing of MV liberation assessments with daily sedation interruptions in protocols for MV liberation is an effective liberation strategy. Girard and co-workers randomized 336 critically ill mechanically ventilated patients to an intervention cohort pairing daily sedation interruption and SBT compared to a cohort that received daily SBT plus usual care for sedation management [26]. Patients receiving the paired management strategy had 3.1 fewer days of MV than the standard care arm. Clinical practice guidelines have provided recommendations to use protocols for MV liberation [5,20,27]. Cochrane reviews have supported the use of protocolized liberation strategies, indicating that protocols reduce the duration of MV, weaning duration, and ICU LOS [28].

## 3. Ventilator Modes and Reducing Ventilator Support

Liberating patients with AHRF or ARDS from invasive MV involves a stepwise process of transitioning the work of breathing from the ventilator to the patient. After intubation, during the ‘acute phase’, the ventilator fully supports patients’ workload of breathing and allows their respiratory muscles to rest. Key aims during this phase are to stabilize patients and ensure adequate gas exchange while preventing ventilator-associated lung injury through the use of lung-protective ventilation strategies, including judicious deep sedation and neuromuscular blockade. As their lungs improve, patients enter into the ‘recovery phase’, wherein their respiratory muscles begin to share in the work of breathing with the ventilator. This phase necessitates diaphragm-protective strategies to avoid overuse injury or underuse atrophy of the diaphragm. The final phase, or ‘weaning phase’, consists of SBTs and is typically followed by either extubation or tracheostomy insertion. Successful transition from ventilator dependence to independence hinges on identifying the right moment for patients to share in the work of breathing, choosing a mode of MV that optimizes patient–ventilator interaction, and adjusting ventilator support levels accordingly.

The first step in this transition involves moving from controlled to assisted ventilation. During the initial week of MV, assist-control is the most prevalent mode of MV used worldwide [29]. With fully controlled ventilation, ventilators trigger all breaths and patients’ diaphragms are passive and rapid deconditioning of the respiratory muscles may occur during this time, increasing the likelihood of prolonged weaning [9,30]. Reducing the set ventilator rate and allowing the patient to trigger ventilator breaths (i.e., assisted ventilation), or switching to a mode of MV wherein patients must trigger every ventilator breath (“partial” or “assist-only” mode) may reduce the risk for diaphragmatic atrophy. However, caution must be exercised, as early use of assisted ventilation for patients with ARDS may result in higher tidal volumes, transpulmonary pressures, and diaphragmatic excursion. In turn, this may increase the risk of developing self-inflicted lung injury and diaphragmatic myotrauma, especially when the respiratory drive and workload are excessive. For patients with AHRF and ARDS, finding the earliest and safest conditions to switch from controlled to assisted ventilation is imperative to protect the lungs and diaphragm.

The optimal time to transition to assisted ventilation has not been studied, and current ARDS guidelines do not specify the optimal conditions for transitioning to assisted ventilation. A target trial emulation using data from the WEAN SAFE (WorldwidE AssessmeNt of Separation of pAtients From ventilatory assistancE) study suggested that early switching (within 1 day of meeting eligibility defined by PaO_2_ to FiO_2_ ratio > 150 mmHg and not receiving neuromuscular blockade) compared to delayed switching was associated with shorter duration of MV without increased mortality [31]. Further evidence, corroborating that early identification and transition to assisted ventilation may be beneficial, comes from the PROMIZING (PROportional assist ventilation for miniMIZING the duration of MV) trial, which included criteria and screening tests for transitioning patients from assist-control to PS and to undergo a SBT [32]. Using this algorithm, 95 (25%) of patients were identified as being ‘ready to extubate’ despite the clinician’s predictions that they would require MV for more than 24 h [7].

Once patients are able to trigger all breaths, a supported mode of ventilation may be used. Options include assist-control, PS, or an adaptive ‘closed loop’ mode of ventilation such as proportional assist ventilation (PAV), neurally adjusted ventilatory assist (NAVA), adaptive support ventilation, or SmartCare™. (Table 1) The NAVIATOR (NAVa In Acute respiraTORy failure) study found that early implementation of NAVA (median 2 days post intubation) resulted in more ventilator-free days and fewer extubation failures compared to patients in the control arm who remained primarily on assist-control [33]. The PROMIZING Study compared proportional assist ventilation plus (PAV+) to PS and found that, while PAV+ did not significantly reduce MV duration when started later (median of 5 days post intubation), it was associated with faster weaning of sedative medications and fewer patient-days testing positive for delirium [34]. PS is the predominant mode used during the recovery phase [35]. Other modes (e.g., PAV and NAVA) are proprietary to specific ventilators.

Clinicians must also consider how to set the level of support for the chosen mode. Two studies provide evidence that gradual weaning of ventilator support may unnecessarily delay successful liberation [9,36]. These studies showed that protocolized SBTs, conducted daily or more frequently through an endotracheal tube or tracheostomy, resulted in shorter duration of MV and higher rates of weaning success compared to a strategy of gradually reducing the level of PS prior to unassisted breathing [9,36]. Although weaning ventilator settings does not seem to be helpful for intubated or tracheostomized patients, protocolized daily SBTs are helpful to identify patients who are ready for ventilator liberation.

Methods for monitoring respiratory mechanics during the transition phase have emerged over the last ten years. Various techniques can be used to assess respiratory drive (e.g., EAdi, P 0.1, mean inspiratory flow, respiratory muscle surface EMG) and respiratory muscle effort (e.g., ΔPes, ΔPocc, PMI, ΔPnose, USdi, ΔCVP, BREF models, and flow index) [37]. Additionally, the consequences of effort (e.g., tidal volume, electrical impedance tomography, dyspnea perception) can be evaluated. Respiratory drive is commonly monitored using the P_0.1_, a parameter that can be readily obtained from most contemporary ventilators. Clinicians target ventilator settings and adjust sedation to maintain P_0.1_ between 2 and 5 cm H_2_O. Conversely, ΔPocc and P_mus_ estimate respiratory effort. Clinicians aim for a ΔPocc that is not more negative than −15 or −20 cm H_2_O (predicts elevated diaphragmatic effort) and titrate ventilator support to maintain P_mus_ between 5 and 10 cm H_2_O. Additionally, clinicians can assess the mechanical consequences of respiratory drive and effort by monitoring tidal volume and maintaining the ΔP_L,dyn_ ≤ 15 cm H_2_O [38].

## 4. Conduct of Spontaneous Breathing Trials

An SBT is a focused assessment of a patient’s ability to breathe without assistance or with low ventilator assistance on inspiration, expiration or both [39]. An SBT is a key component of a multifaceted and dynamic process to evaluate a patient’s ability to be liberated from ventilators. Whereas SBTs are conducted to assess patients’ readiness for ventilator liberation, extubation assessments assess patients’ ability to be liberated from the need for an airway. A large observational study of MV discontinuation practices identified that 18.2% of patients failed an initial SBT. Both initial failed (versus successful) SBTs and initial SBTs that occurred later (versus earlier) during the ICU course have been associated with worse clinical outcomes [35]. Whereas some patients will pass an initial SBT and be extubated, others may require more than one SBT before extubation, and some may not be extubated.

**Figure 1:**
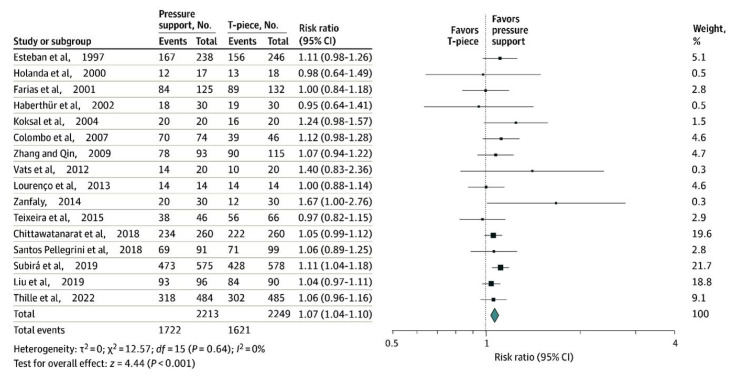
Association of pressure support compared with T-piece spontaneous breathing trials on successful extubation. CI = confidence interval [40,41,45,46,47,48,49,50,51,52,53,54,55,56,57,58]. Reprinted from Ref. [43].

The optimal SBT technique for clinicians to use remains a matter of considerable debate with conflicting results in RCTs and meta-analyses. Among 1153 patients receiving MV, 30 min PS SBTs compared to 2 h T-piece SBTs resulted in significantly higher rates of successful extubation [40]. Conversely, in 969 patients at high risk for extubation failure, Thille and coworkers found that PS SBTs did not result in significantly more ventilator-free days at day 28, compared to T-piece SBTs [41]. A physiologic meta-analysis of one parallel group trial, eight randomized crossover and seven nonrandomized crossover studies found that T-piece SBTs may more closely replicate the work of breathing required after extubation [42]. Conversely, a pairwise meta-analysis of 40 RCTs (n = 6716) that compared alternative SBT techniques identified that, although patients were not more likely to pass PS (versus T-piece) SBTs [risk ratio (RR), 1.04 (95% CI; 0.97–1.11; *p* = 0.31 I^2^ = 73%], unless a single trial accounting for all heterogeneity was excluded [RR 1.09; 95% CI, 1.06–1.12; *p* < 0.001; I^2^ = 0%] (moderate quality evidence), they were significantly more likely to be successfully extubated with PS (versus T-piece) SBTs [RR 1.07 (95% CI; 1.04, 1.10; *p* < 0.001, I^2^ = 0%] (moderate quality evidence) (Figure 1) [43]. A subsequent network meta-analysis found that compared to T-piece SBTs, initial successful SBT rates were increased with PS [risk ratio (RR) 1.08, 95% confidence interval (CI) (1.05–1.11)], PS/automatic tube compensation (ATC) [1.12 (1.01–1.25)], high flow nasal cannulae (HFNC) [1.07 (1.00–1.13) (all moderate certainty), and ATC [RR 1.11, (1.03–1.20); low certainty] SBTs [44]. Compared to T-piece SBTs, successful extubation rates were increased with PS [RR 1.06, (1.03–1.09); high certainty], ATC [RR 1.13, (1.05–1.21); moderate certainty], and HFNC [RR 1.06, (1.02–1.11); high certainty] SBTs [44]. Although certainty was low, there did not appear to be a difference in reintubation rates with PS (versus. T-piece) SBTs; however, increased reintubation rates were identified with PS versus HFNC [RR 2.84, (1.61–5.03); moderate certainty] and ATC versus HFNC [RR 2.95 (1.57–5.56); moderate certainty] SBTs in a small number of trials [44].

Trials reporting SBTs have been hampered by design issues including inclusion of heterogeneous patients, mostly with high pre-test probability of success, variable use of protocolized screening and intervention application (applied to the initial SBT only or serially until a trial end-point is achieved), and heterogeneous outcomes reporting. Whereas SBT techniques that use pressure augmentation may lead to false positive results and contribute to a failed extubation, SBTs conducted without pressure augmentation may lead to false negative results and expose patients to continued invasive MV. Though no single SBT technique may be ideally suited for all diverse patients (e.g., neuromuscular weakness, pulmonary edema, chronic obstructive pulmonary disease) who require ventilator liberation, the use of inspiratory pressure augmentation during SBTs appears to increase the rate of successful extubation without increasing the rate of reintubation. Based on the best available evidence, a clinical practice recommended that patients have an initial SBT performed with inspiratory pressure augmentation [20].

Typically, SBTs are 30 to 120 min in duration. Only four RCTs have been conducted to compare SBTs of different durations either alone or in combination with other interventions. Of these, three RCTs compared SBTs of 30 to 120 min and one RCT compared 20 to 120 min [40,59,60,61]. Although 30 min SBTs may be reasonable for most patients, we do not know which patients may benefit from a longer SBT. No trial comparing alternative SBT durations was conducted to assess equivalency, and guidelines do not comment on SBT duration.

Conflicting data exist regarding ventilator reconnection after successful SBT completion. Coudroy et al. identified significant alveolar derecruitment at the end of an SBT, especially with T-piece (versus PS) SBTs, which was ameliorated by reconnecting patients to the ventilator for an hour [62]. One trial did not find a benefit to ventilator reconnection [63], while another trial [64] found a significant reduction in reintubation rate for patients who were invasively ventilated for more than 12 h.

## 5. Extubation

Several reviews have summarized predictors of weaning and extubation outcomes. In a 2018 systematic review, Baptistella and colleagues reviewed 43 articles (n = 7989) to identify 56 different parameters used to predict weaning and extubation outcomes. They identified that the RSBI was the most frequently investigated predictor (15 studies), followed by age and maximum inspiratory pressure (7 studies). The remaining 53 parameters were identified in fewer than 6 studies [65]. In 2021, Torrini and coworkers conducted a systematic review to explore factors associated with extubation failure in patients who passed a SBT and underwent planned extubation; they found 26 factors in 3 domains (comorbidities, acute disease severity, characteristics at the time of extubation) significantly associated with extubation failure and focused predominantly on respiratory and neurologic systems [66]. Using Bayesian multivariable meta-analysis, they identified 12 factors significantly associated with extubation failure (age, history of cardiac disease, history of respiratory disease, Simplified Acute Physiologic Score II score, pneumonia, duration of ventilation, heart rate, RSBI, negative inspiratory force, lower PaO_2_/FiO_2_ ratio, lower hemoglobin level and lower Glasgow Coma Scale (GCS) [67]), with GCS having the strongest association with extubation outcome [66]. In a systematic review of predictors of weaning failure, Sterr and colleagues identified 145 predictors in 140 studies (122 prospective, 18 retrospective) that could be categorized into 4 clusters: physiologic parameters (n = 61), scores and indices (n = 53), imaging procedures (n = 22), and machine learning models (n = 9) [68]. Finally, a systematic review of ten systematic reviews examining the diagnostic accuracy of 23 readiness tests conducted before, during or after SBTs identified lung ultrasound score, diaphragmatic RSBI, venous oxygen saturation, and brain natriuretic peptide as having high-to-moderate sensitivity and specificity for weaning failure [69].

Since numerous factors have been associated with weaning and extubation failure, attention has drawn away from single physiologic predictors to clinical scores and newer artificial intelligence systems that include multiple parameters [65,66]. Several indices, comprising two or more parameters, have been proposed, including the RSBI [7], CROP index [7], Core index [70], Timed Inspiratory Index (TIE) [71], respiratory rate variability [72] and diaphragmatic RSBI [73], to predict weaning and extubation outcomes. To aid prediction, parameters have also been combined into scores, including the Burns Weaning Assessment Program (BWAP) [74] and modified BWAP [75]; Weaning index [76]; original and new Integrative Weaning Index [77,78]; Integrative Pulmonary Index [79]; Extubation Prediction Score (ExPreS) score [80]; heart rate, acidosis, consciousness, oxygenation, and respiratory rate (HACOR) score [81]; and the COBRE-US model [82]. Newer approaches have utilized neural networks [83,84,85], convolutional neural networks [86], artificial intelligence [87], and machine learning [88,89,90,91] to predict weaning and extubation outcomes. Few indices or scores have been formally evaluated in RCTs. In 2006, Tanios et al. showed no difference in extubation failure rate and a longer duration of weaning for 153 patients who had the RSBI measured and used (at a threshold of 105 breaths/min/L) in the decision to wean compared to 151 patients who had the RSBI measured with no incorporation of the RSBI into the decision to wean [92]. In an RCT of 540 patients, Baptistella found that the use of the ExPreS score reduced the rate of reintubation within 48 h of extubation in per-protocol, but not in intention-to-treat, analyses. Rescue use of NIV and dependence on NIV after extubation for at least 48 h were considered to represent extubation failure in this trial [93]. Finally, specific airway predictors, largely reflecting airway patency and cough strength, have been investigated, including the GCS, cough strength [94], cuff-leak test [95], and peak cough flow [96].

Newer techniques such as lung–diaphragm–abdominal ultrasound and EIT provide insights into the mechanisms underlying extubation failure. Lung ultrasound permits bedside assessment of pulmonary edema, atelectasis, and consolidation [97]. Conversely, diaphragm ultrasound provides a window into the capacity of the primary inspiratory muscle to sustain spontaneous breathing [98]. A prospective observational study conducted in 50 mechanically ventilated, weaning-ready patients evaluated diaphragm excursion and diaphragmatic thickening fraction alongside conventional weaning parameters during SBTs [99]. Patients who failed SBTs had significantly lower diaphragm excursion and thickening fraction compared with those who succeeded. In a larger cohort study of 128 patients undergoing SBTs with planned extubation, diaphragmatic thickening fraction was significantly higher in the success group (mean of 42%) compared to the failure group (mean of 20%) [100]. These findings align with the conclusion of a systematic review that reported an association between diaphragm ultrasound parameters and weaning outcomes but highlighted substantial heterogeneity, variable thresholds, and dependence on measurement timing and ventilator settings [101]. EIT provides bedside assessment of regional ventilation distribution and changes in lung impedance during ventilator support transitions [102]. Wang and colleagues showed that higher pre-SBT global impedance and higher impedance in Region of Interest 2 during SBTs were associated with weaning success, whereas lower values were observed in patients who failed liberation from MV [103]. Phoophiboon et al. showed that a pronounced ventral-to-dorsal difference in ventilation distribution with EIT during SBTs may identify patients at risk of liberation failure [104]. Consequently, EIT may aid in elucidating underlying lung reserve and susceptibility to derecruitment during unassisted breathing and detect unfavorable ventilation patterns that emerge during the withdrawal of positive pressure.

More recently, abdominal muscle ultrasound has been used to evaluate expiratory muscle function and cough effectiveness in extubation assessments. Abdominal muscles play a key role in generating effective cough and clearing secretions, mechanisms that are critical for maintaining airway patency after extubation. Ultrasound assessment of abdominal muscle thickening has been shown to correlate with airway pressure generated during expiratory efforts and to be feasible and moderately reproducible in mechanically ventilated patients [105]. Importantly, among patients who passed an SBT, reduced abdominal muscle thickening during cough was associated with a high risk of liberation failure. These findings support the concept of a cough-ineffective phenotype, distinct from inspiratory muscle weakness or lung derecruitment, in which extubation failure arises from impaired secretion clearance rather than ventilatory insufficiency.

Extubation failure is rarely the consequence of a single physiological abnormality. Rather, it is typically multifactorial, reflecting the interaction of multiple mechanisms that may coexist, including an imbalance between respiratory system load and capacity, ineffective cough, secretion burden, and other factors. As a result, traditional weaning indices and SBTs that largely capture global respiratory patterns may fail to identify patients at risk for extubation failure. Techniques such as EIT and lung, diaphragm, and abdominal muscle ultrasound may provide mechanistic insights into the pathways leading to extubation failure and improve risk stratification. The potential role of these modalities is twofold. First, they may help identify patients at higher risk of extubation failure or enhance the prognostic performance of existing indices. These patients may benefit from closer post-extubation monitoring or early application of noninvasive respiratory support strategies. Second, they may help characterize the dominant phenotype underlying the risk for extubation failure and inform targeted preventive or therapeutic strategies. As such, these newer modalities offer complementary, mechanism-based insights into extubation failure. By identifying patients at increased risk for extubation failure and clarifying the physiological phenotype driving that risk, these newer modalities offer complementary, mechanism-based insights into extubation failure.

## 6. Post-Extubation Use of Noninvasive Respiratory Support Strategies

Noninvasive modalities of respiratory support, including HFNC and bilevel NIV, may mitigate the risk of extubation failure. A large systematic review and network meta-analysis of 36 RCTs and 6806 patients found that, compared with conventional oxygen therapy (COT), both HFNC and NIV probably reduce the rate of reintubation (moderate-certainty evidence) [106] (Table 2). The magnitude of benefit was greatest among patients with a higher baseline risk for reintubation. The number needed to treat (NNT) to prevent one reintubation was 60 among patients with a baseline reintubation risk of 5%, compared with an NNT of 11 among those with a baseline risk of 40%. Importantly, these benefits were observed only when noninvasive respiratory support strategies were applied prophylactically rather than as rescue therapy following clinical deterioration [106]. Despite reductions in reintubation rates, neither HFNC nor NIV were associated with a reduction in short-term mortality (moderate-certainty evidence) [106]. However, noninvasive respiratory support was associated with a lower incidence of ventilator-associated pneumonia (moderate-certainty evidence), likely mediated by reduced rates of reintubation.

In direct comparisons of HFNC and NIV, low-certainty evidence suggested no clear superiority of one modality over the other for preventing reintubation in the general critically ill population [106]. However, NIV may confer additional benefit in patients with obesity, particularly when used in combination with HFNC. This finding is supported by a meta-analysis of seven RCTs including 1933 patients with obesity, which demonstrated that NIV combined with HFNC probably reduces 7-day reintubation rates compared with COT or HFNC alone (moderate-certainty evidence) and was also associated with reduced 28-day mortality [107].

**Figure 2:**
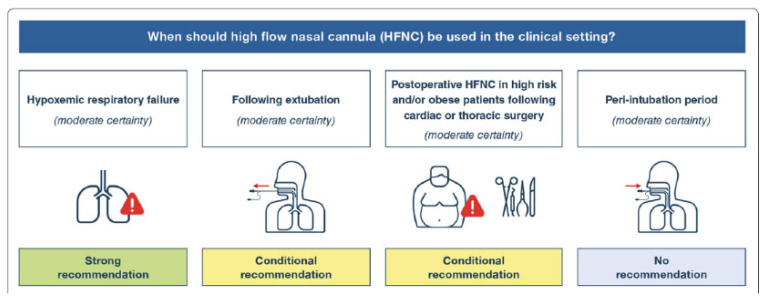
Use of high flow nasal cannulae (HFNC) in the clinical setting. Rochwerg B, et al [108]. Intensive Care Medicine, 2020, Springer Nature. Reproduced with permission from Springer Nature.

Guidelines from the European Society of Intensive Care Medicine (ESICM) and the European Respiratory Society (ERS) provide conditional or weak recommendations for the use of HFNC over COT in patients intubated for more than 24 h who have high-risk features, as well as in nonsurgical patients following extubation [108,109] (Figure 2). Guidelines also suggest the use of NIV for patients who would otherwise be extubated directly to NIV [108] and are considered to be at high-risk for extubation failure [109].

Several important uncertainties remain to guide the use of HFNC and NIV after extubation. Definitions for ‘high-risk’ patients vary substantially across studies. Specific populations, beyond those with obesity, that derive the greatest benefit from prophylactic NIV and HFNC remain to be characterized. The potential merits of newer interfaces and technologies, including helmet-based NIV or the newer asymmetric HFNC, have not been adequately evaluated in the post-extubation period. Well-designed trials are needed to define optimal patient selection, modality choice, and implementation strategies for noninvasive respiratory support devices.

## Figures and Tables

**Table 1:** Closed-loop modes of ventilation used during liberation from ventilation.

Newer Modes	Main Feature
Proportional Assist Ventilation	Airway pressure is amplified according to respiratory mechanics and the set level of assistance to be proportional to the instantaneous effort of the patient.
Neurally Adjusted Ventilatory Assist	Measures the electrical activity of the diaphragm (EAdi) and provides positive pressure synchronously and in proportion to the amplitude of the EAdi signal.
Adaptive Support Ventilation	Targets a desired minute ventilation by providing the optimal combination of tidal volume and respiratory rate according to Otis’ equation (calculates an ideal respiratory rate that is associated with the least energy expenditure by considering minute ventilation, dead space and the expiratory time constant of the respiratory system). Algorithm calculates the expiratory time constant and adjusts the I:E ratio and inspiratory pressure to reduce work of breathing.
SmartCare	Algorithm adjusts the level of pressure support provided according to 8 respiratory diagnoses that aim to achieve a respiratory comfort zone based on respiratory rate, tidal volume and end-tidal carbon dioxide. It automatically conducts spontaneous breathing trials once predetermined settings are achieved.

**Table 2:** Network and absolute estimates evaluating the efficacy of the interventions for prevention of reintubation in critically ill adults.

Comparison	Network Odds Ratio (95% CI)	Absolute Risk Difference (95% CI)	Number Needed to Treat	GRADE
NIPPV vs. conventional oxygen	0.65 (0.52–0.82)	–5.18 (–8.09 to –2.26)	20 (13 to 45)	Moderate ^a^
HFNC vs. conventional oxygen	0.63 (0.45–0.87)	–3.84 (–6.7 to –0.98)	26 (15 to 102)	Moderate ^a^
NIPPV vs. HFNC	1.04 (0.78–1.38)	–1.34 (–4.4 to 1.72)	N/A	Low ^a,b^
HFNC + NIPPV vs. conventional oxygen	0.38 (0.19–0.74)	–10.25 (–18.49 to –2.01)	10 (6 to 50)	Moderate ^a^
HFNC + NIPPV vs. NIPPV	0.58 (0.3–1.11)	–5.07 (–13.38 to 3.24)	N/A	Low ^a,b^
HFNC + NIPPV vs. HFNC	0.6 (0.33–1.08)	–6.41 (–14.13 to 1.31)	N/A	Low ^a,b^

Abbreviations: NIPPV: noninvasive positive pressure ventilation, HFN: high-flow nasal cannula, GRADE: grading of recommendations assessment, development, and evaluation, OR: odds ratio, CI: confidence interval. ^a^ Lowered for risk of bias. ^b^ Lowered one level for imprecision as CIs don’t exclude harms. Reprinted from Ref. [107].

## Data Availability

No new data were created or analyzed in this study.
